# The clinical features of the overlap between COPD and asthma

**DOI:** 10.1186/1465-9921-12-127

**Published:** 2011-09-27

**Authors:** Megan Hardin, Edwin K Silverman, R Graham Barr, Nadia N Hansel, Joyce D Schroeder, Barry J Make, James D Crapo, Craig P Hersh

**Affiliations:** 1 Channing Laboratory, Brigham and Women's Hospital, Harvard Medical School, Boston, MA, USA; 2 Division of Pulmonary and Critical Care Medicine, Brigham and Women's Hospital, Harvard Medical School, Boston, MA, USA; 3 Department of Medicine, College of Physicians and Surgeons, Columbia University, New York, NY, USA; 4 Department of Pulmonary and Critical Care Medicine, Johns Hopkins University, Baltimore, MD, USA; 5 Division of Pulmonary Sciences and Critical Care Medicine, National Jewish Health, Denver, CO, USA

**Keywords:** Airway hyperresponsiveness, asthma, Chronic obstructive pulmonary disease, emphysema, Exacerbation, Gas-trapping

## Abstract

**Background:**

The coexistence of COPD and asthma is widely recognized but has not been well described. This study characterizes clinical features, spirometry, and chest CT scans of smoking subjects with both COPD and asthma.

**Methods:**

We performed a cross-sectional study comparing subjects with COPD and asthma to subjects with COPD alone in the COPDGene Study.

**Results:**

119 (13%) of 915 subjects with COPD reported a history of physician-diagnosed asthma. These subjects were younger (61.3 vs 64.7 years old, p = 0.0001) with lower lifetime smoking intensity (43.7 vs 55.1 pack years, p = 0.0001). More African-Americans reported a history of asthma (33.6% vs 15.6%, p < 0.0001). Subjects with COPD and asthma demonstrated worse disease-related quality of life, were more likely to have had a severe COPD exacerbation in the past year, and were more likely to experience frequent exacerbations (OR 3.55 [2.19, 5.75], p < 0.0001). Subjects with COPD and asthma demonstrated greater gas-trapping on chest CT. There were no differences in spirometry or CT measurements of emphysema or airway wall thickness.

**Conclusion:**

Subjects with COPD and asthma represent a relevant clinical population, with worse health-related quality of life. They experience more frequent and severe respiratory exacerbations despite younger age and reduced lifetime smoking history.

**Trial registration:**

ClinicalTrials.gov: NCT00608764

## Background

Chronic Obstructive Pulmonary Disease (COPD) affects over 10 million Americans. In the United States, COPD is the third leading cause of death [1] and is responsible for over $15 billion in annual healthcare costs [2]. World-wide, COPD is one of the few conditions in which mortality is rising, and it is estimated to become the third leading cause of death by 2020[3,4]. More than 40% of patients with COPD will additionally report a history of asthma, and this dual-diagnosis increases with age [5,6].

There is increasing evidence that patients who have COPD and asthma experience more rapid disease progression than those with either disease alone. Airway hyperresponsiveness (AHR) and the diagnosis of asthma have been associated with greater decline in FEV_1 _in both smokers and nonsmokers [7-9] and asthma has been recognized as a risk factor for COPD [10]. The presence of AHR in patients with COPD has been associated with an increase in COPD exacerbations and overall mortality [11], and the coexistence of asthma and COPD is associated with increased co-morbidities and health-care utilization [12,13].

Despite these known interactions between COPD and asthma, the clinical aspects of this overlap population have not been well described. In fact, the dual-diagnosis of COPD and asthma is often an exclusion criterion for participation in studies investigating either disease alone [14]. The aim of this study was to examine the clinical, physiologic and chest CT scan features of subjects with the overlapping diagnoses of COPD and asthma in comparison to subjects with COPD alone. We hypothesized that subjects with COPD and asthma would have markers of more severe disease, including greater exacerbation frequency and worse quality of life scores. We analyzed subjects in the Genetic Epidemiology of COPD Study (COPDGene) study, a large case-control study of COPD for which asthma was not an exclusion criterion.

## Methods

### Study Subjects and Procedures

We performed a cross-sectional observational study among smoking subjects to investigate unique clinical characteristics of subjects with COPD and asthma compared to subjects with COPD alone, using data complete through November 2010 from the first 2500 subjects enrolled in the COPDGene Study. COPDGene is a multicenter study that aims to improve classification of COPD phenotypes as well as determine the genetic background of this disease. Study details including enrollment procedures and phenotyping have been described [15] and all protocols and questionnaires are available at http://www.copdgene.org. Briefly, all subjects had at least a 10 pack-year smoking history and were self-identified non-Hispanic whites or non-Hispanic African Americans between 45-80 years old. Exclusion criteria included pregnancy, active cancer, and respiratory disorders other than asthma. 21 clinical centers participated in this study and IRB approval was obtained for all participating centers. All subjects signed informed consent prior to enrollment.

All study subjects completed standardized questionnaires regarding medical history and respiratory symptoms. Measures of disease severity included health-related quality of life as determined by the St George's Respiratory Questionnaire (SGRQ) [16], predicted mortality using the BODE index (Body mass index, airflow obstruction, dyspnea and exercise capacity index) [17], history of hay fever, respiratory medication use, and history of exacerbations of COPD. Severe exacerbations were defined as respiratory exacerbations in the year prior to enrollment that resulted in presentation to a hospital or emergency department. Frequent exacerbators were defined as those subjects who experienced 2 or more respiratory exacerbations that required antibiotics, steroids, or presentation to a physician or hospital in the year prior to enrollment. This definition was chosen based on recent data demonstrating that a history of two or more exacerbations in the prior year is a stable phenotype in COPD, which can predict future frequent exacerbations [18]. Number of exacerbations was defined as the absolute number of exacerbations each subject experienced in the year prior to enrollment. All subjects performed standardized pre-and post-bronchodilator spirometry (180 mcg [2 puffs] of albuterol), in accordance with ATS guidelines [19]. All subjects completed a standardized 6-minute walk test (6MWT) [20].

The presence of emphysema and gas trapping were determined in all subjects by computational analysis of chest CT scans using 3D SLICER software. Emphysema was measured as the percentage of lung with attenuation values less than or equal to -950HU on inspiratory images, and gas trapping was measured as the percentage of lung less than or equal to -856HU on expiratory images [21,22]. Airway wall thickness was measured using VIDA software (VIDA diagnostics, Iowa City, IA) by determining the square root wall area of a hypothetical airway of 10 mm internal perimeter (Pi10) [23].

### Statistical analysis

The primary analysis included all subjects with GOLD stage 2 or higher COPD (post-bronchodilator FEV_1_/FVC < 0.7 and FEV_1 _< 80% predicted). Asthma was defined by subject report of a physician-diagnosis of asthma before the age of 40. Subjects with a diagnosis of asthma after the age of 40 or at an unknown age were excluded from this analysis. This age exclusion was chosen to improve the accuracy of the asthma diagnosis. Exacerbation frequency was dichotomized as zero COPD exacerbations compared with two or more COPD exacerbations in the year prior to study enrollment. All spirometry data were adjusted for age, race, gender, height, and pack-years of smoking [24].

Contingency tables and Fisher's exact test were performed for all univariate comparisons. Continuous variables were compared using nonparametric analysis with the Wilcoxon rank sum test. Linear and logistic regression models were performed to adjust for potentially confounding variables chosen based on known clinical confounders, in the following manner: BODE score, SGRQ, and COPD exacerbations were adjusted for age, gender, pack-years of smoking and race. Chest CT scan variables were adjusted for age, gender, race, BMI, smoking history, as well as type of CT scanner used. A logistic regression model was created to assess potential contributing factors to the diagnosis of asthma in subjects who have COPD.

## Results

**Figure 1:**
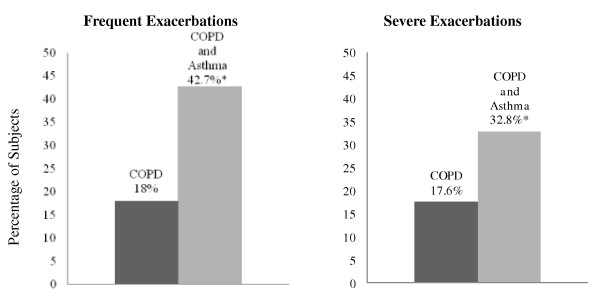
**Exacerbations: Percentage of frequent and severe exacerbations among subjects with COPD compared to subjects with COPD and asthma**. *p < 0.0001 for the difference between COPD and COPD with asthma.

We examined the first 2500 subjects from the COPDGene study. Of these, 1059 subjects had COPD defined as GOLD stage 2-4. We excluded 144 subjects who did not provide information about their asthma history, leaving 915 subjects for analysis. Out of 915 COPD subjects, 119 (13%) reported a physician's diagnosis of asthma prior to the age of 40. In comparison to subjects with COPD alone, subjects with COPD and asthma were younger (61.3 vs 64.7 years old, p = 0.0001) and had fewer pack-years of smoking (43.7 vs 55.1, p < 0.0001) (Table 1). More subjects with COPD and asthma additionally reported a history of hay fever than subjects with COPD alone. There was no significant difference in gender among the groups or in current smoking status. There were more African-American COPD subjects who reported a history of asthma, and there were more subjects in this group who were frequent exacerbators in the year prior to enrollment (Figure 1). In an unadjusted analysis, more subjects with COPD and asthma were currently taking inhaled corticosteroids in comparison to those with COPD alone (p < 0.0001); however, when adjusted for FEV_1_, this difference was no longer significant (Table 1). A secondary analysis performed without the use of the age restriction in the definition of asthma demonstrated consistent findings. 1026 subjects were included and 223 (21.7%) reported a history of asthma. In contrast to the restricted analysis, more women were found to additionally report a history of asthma (44% with COPD only compared to 56% with COPD and asthma, p = 0.012).

**Table 1:** Characteristics of COPD cases (GOLD stage 2 or greater) with and without physician-diagnosed asthma.

	COPD Only	COPD and Asthma	p-value
Total Subjects	796	119	--

Race:			
Non-Hispanic white	672 (84.4)	79 (66.4)	< 0.0001
African American	124 (15.6)	40 (33.6)	

Gender			
Male	423 (53.1)	61 (51.3)	0.77
Female	373 (46.9)	58 (48.7)	

Age, years	64.7 (8.2)	61.3 (8.9)	0.0001*

Pack-years of smoking	55.1 (27.3)	43.7 (20.7)	< 0.0001*

Current smoker	270 (34.2)	46 (38.7)	0.35

BMI kg/m^2^	27.8 (6.0)	28.1 (6.7)	0.87*

GOLD Stage			
2	408 (51.3)	61 (51.3)	0.99**
3	251 (31.5)	38 (31.9)	
4	137 (17.2)	20 (16.8)	

BODE Index	3.0 (2.1)	3.2 (1.9)	0.28*

SGRQ	38.6(20.5)	44.0 (21.9)	0.0075*

6MWT (ft)	1140.2 (431.9)	1158.8 (430.1)	0.78*

Severe exacerbations	140 (17.6)	39 (32.8)	0.0003

Frequent exacerbations	114 (18.0)	41 (42.7)	< 0.0001

Hay fever	204 (27.8)	65 (57.0)	< 0.0001

Inhaled corticosteroid use	387 (49.4)	80 (69.0)	0.25***

FEV_1 _% predicted	49.4 (18.4)	49.2 (17.5)	0.85*

FVC % predicted	76.6 (17.9)	78.3 (17.3)	0.23*

FEV_1_/FVC	0.48 (0.13)	0.48 (0.12)	0.62*

Presence of bronchodilator response, ATS definition	270 (36.0)	46 (41.1)	0.30

Post bronchodilator % change in FEV_1_	8.7 (12.1)	10.2 (12.5)	0.33*

Percent Emphysema, -950HU	15.8 (13.5)	13.7 (12.3)	0.18*

Percent gas-trapping, -856HU	42.1 (20.3)	43.1 (20.5)	0.69*

Airway wall thickness Pi10	3.78 (0.12)	3.80 (0.14)	0.34*

Values are presented as mean (SD) or N (%).

Abbreviations and definitions: COPD = chronic obstructive pulmonary disease; Hay fever = self report of a history of hay fever; BODE index = body mass index, airflow obstruction, dyspnea and exercise capacity index; SGRQ = St George's Respiratory Questionnaire; 6MWT = 6 minute walk test; Severe Exacerbations = presence of COPD exacerbation resulting in presentation to emergency room or hospital admission in year prior to presentation; Frequent exacerbations = two or more COPD exacerbations in the year prior to enrollment; FEV_1 _= forced expiratory volume in 1 second; FVC = forced vital capacity; Bronchodilator response = an increase in FEV_1 _of > 12% and 200 ml after administrator of bronchodilator; Pi10 = square root wall area of a hypothetical airway of 10 mm internal perimeter. Data compared using Fisher's exact test unless otherwise stated. *Wilcoxon rank sum analysis. **Chi-square analysis. ***p value adjusted for FEV_1_.

Table 2 shows that subjects with COPD and asthma demonstrated increased disease severity based on multiple measures. In multivariate comparisons adjusting for potential confounders including age, gender, pack-years of smoking and race, subjects with COPD and asthma had worse health-related quality of life, with a clinically significant 5.2 point higher SGRQ score (p = 0.009). They were nearly twice as likely to experience severe respiratory exacerbations in the year prior to enrollment and were more than three times as likely to be frequent exacerbators in the year prior to enrollment. There was no significant difference in BODE index, 6MWT distance and spirometry, including FEV_1_, FEV_1_/FVC and bronchodilator response. Adjusting for BMI, age, gender, pack-years and race, subjects with COPD and asthma demonstrated greater gas trapping on expiratory CT scans as well as greater subsegmental wall area as measured by the parameter percent wall area (66.2% vs 65.5% subsegmental wall area, p = 0.013) on inspiratory CT scans. There was no difference in percent emphysema or airway wall thickness (Pi10) on inspiratory chest CT scans.

**Table 2:** Association of concurrent asthma diagnosis and COPD severity outcomes in COPD subjects.

Outcome	Additional Covariates	β(SE)/OR(CI)	p-value
BODE Index	age, gender, pack-years and race	0.29 (0.21)	0.17

SGRQ	age, gender, pack-years and race	5.2 (2.0)	0.009

6MWT, ft	age, gender, pack-years and race	31.6 (40.5)	0.43

Bronchodilator Response	age, gender, pack-years, and race	1.40[0.92, 2.13]	0.12

Severe exacerbations	age, gender, pack-years and race	1.93 [1.24, 3.02]	0.004

Frequent exacerbations	age, gender, pack-years and race	3.55 [2.19, 5.75]	< 0.0001

Number of exacerbations	age, gender, pack-years and race	0.68 (0.12)	< 0.0001

Percent Emphysema, -950HU	BMI, age, gender, pack-years, race and CT scanner*	-0.45 (1.23)	0.71

Percent gas-trapping, -856HU	BMI, age, gender, pack-years, race and CT scanner	4.31 (1.88)	0.02

Airway wall thickness Pi10	BMI, age, gender, pack-years, race and CT scanner	0.016 (0.013)	0.21

Abbreviations and definitions: COPD = chronic obstructive pulmonary disease; BODE index = body mass index, airflow obstruction, dyspnea and exercise capacity index; SGRQ = St George's Respiratory Questionnaire; 6MWT = 6 minute walk test; Severe Exacerbations = presence of COPD exacerbation resulting in presentation to emergency room or hospital admission in year prior to presentation; Frequent exacerbations = two or more COPD exacerbations in the year prior to enrollment; Number of exacerbations = number of COPD exacerbations in the year prior to enrollment; Pi10 = square root wall area of a hypothetical airway of 10 mm internal perimeter. *Siemens 64 Sensation scanners gave aberrant lung density measurements and therefore this scanner type was adjusted as a covariate.

In a logistic regression analysis examining predictors for the presence of asthma in subjects with COPD (Table 3), African-American race was associated with a two-fold increase in the risk of diagnosis of asthma. The diagnosis of asthma was less likely with increasing age or for every ten pack-years of smoking history.

**Table 3:** Multivariate logistic regression for predictors of asthma among subjects with COPD:

Variable	OR [CI]	p-value
African-American race	2.05 [1.31, 3.21]	0.002

Female gender	0.94 [0.63, 1.40]	0.76

Older age (per decade)	0.76 [0.60, 0.97]	0.03

Pack years of smoking (per 10 pack years)	0.86 [0.78, 0.95]	0.002

In a stratified analysis in which the effect of asthma was examined separately among the subjects with moderate COPD (GOLD stage 2) and the subjects with severe and very severe COPD (GOLD stages 3 and 4), there was no significant difference in spirometry data based on asthma diagnosis. Among subjects with moderate COPD (GOLD stage 2), subjects with asthma and COPD overlap demonstrated greater percent gas-trapping on expiratory chest CT scans compared to those with COPD alone (6.4 +/- 2.2%, p = 0.003). This difference was not seen among subjects with severe COPD (-1.5 +/- 2.0%, p = 0.45). Among subjects with severe COPD there was a trend towards less percent emphysema on inspiratory CT scan in subjects with COPD and asthma compared to those with COPD alone (18.8% vs 23.0%, p = 0.05). The presence of asthma was significantly associated with an increase in respiratory exacerbations regardless of COPD severity (among the moderate group: OR 3.60 [1.72, 7.54], p = 0.007; among the severe COPD group: OR 3.55 [1.76, 7.15], p = 0.0004).

## Discussion

In the COPDGene Study, we demonstrate that subjects with COPD and asthma have distinct and clinically-relevant characteristics. These subjects are more likely to be younger, African-American, and have less smoking history. However their lung function is similar to that of subjects with COPD alone. Despite this, they have worse health-related quality of life and are more likely to have frequent and severe respiratory exacerbations, a marker for more severe disease overall.

This is the first study to specifically link the presence of asthma to more frequent respiratory exacerbations in subjects with COPD. Recent studies have demonstrated that the presence of frequent exacerbations appears to be a stable phenotype of COPD that exists across all levels of lung function and is identifiable by the presence of 2 or more exacerbations per year. In our study, the presence of frequent exacerbations was more common in subjects with COPD and asthma across all GOLD stages. Our findings are consistent with and extend the findings of a previous study demonstrating that patients with COPD and asthma have greater healthcare utilization related to pulmonary disease, with five times greater health care costs when compared to subjects with either disease alone [12]. These results have implications for disease management as the presence of increased COPD exacerbations has been associated with worse health-related quality of life as well as overall mortality [25,26]. The coexistence of asthma and COPD may identify a group of patients that are more likely to include frequent exacerbators and management of these patients should be directed towards exacerbation prevention.

This study is one of the first large studies to describe the chest CT scan findings in subjects with both COPD and asthma. These subjects demonstrated more gas-trapping on expiratory chest CT scans compared to subjects with COPD alone. This is consistent with prior CT imaging studies demonstrating an increase in gas-trapping among subjects with asthma and may reflect an increase in small airway disease in this group [27]. There was no difference in emphysema or airway wall thickness between COPD subjects with and without asthma. When we restricted our analysis to subjects with moderate COPD, who may be expected to have the most variability in degree of emphysema, we did not see a difference in the percentage of emphysema between those subjects with COPD and those with COPD and asthma. Taken as a whole, these findings could suggest that airway inflammation rather than parenchymal destruction plays a greater role in the decrease in pulmonary function in subjects with COPD and asthma.

We additionally demonstrate a greater portion of subjects with asthma in the African-American population. These findings would suggest that clinicians should be more vigilant to screen for lung function decline in African-American patients with asthma, and similarly should be alert for an asthmatic component to African American subjects with COPD. This latter suggestion is supported by our multivariate analysis for clinical predictors of the diagnosis of asthma, in which we found that African-Americans with COPD were twice as likely to have asthma compared to white subjects with COPD. These findings could reflect a bias towards defining lung function decline in African American populations as asthma in comparison to COPD. These findings could also reflect the greater prevalence of asthma in this population [28]. Although prior studies have demonstrated that clinicians are less likely to diagnose COPD in women than in men [29], when examining subjects with a history of asthma diagnosed before age 40, we did not see a greater percentage of female subjects with asthma in addition to COPD. Of interest, when we examined our study population without the age restriction, we did see more women than men reporting a history of asthma.

Our study has several limitations. This is a cross-sectional study and therefore we are not able to address how COPD and asthma interact to produce this more severe disease phenotype. This would require a cohort analysis with decades of follow-up time, a potentially expensive and prolonged investigation. We were limited by our definition of asthma which included those subjects with a self report of a physician diagnosis of asthma. Self-report of physician diagnosis of asthma has been used previously in many large trials, and the percentage of subjects with asthma in COPDGene was similar to other large cohorts of subjects with COPD. More objective methods to diagnose asthma may be limited in subjects with COPD. Methacholine challenge testing in COPD subjects with FEV1 < 70% predicted has limited safety experience and can often be positive in the absence of asthma [30]. Based on self-report of physician diagnosis of asthma, we were able to identify a unique clinical phenotype among subjects with COPD. In addition, subjects with asthma demonstrated a greater incidence of atopy with more frequent occurrence of hay fever. The absence of a difference in bronchodilator responsiveness between subjects with and without asthma may reflect the overlapping nature of these diseases. Alternatively, one potential explanation is that subjects were not required to withhold their regular bronchodilator medication before spirometry testing. In order to further clarify the nature of respiratory disease between our two study populations, we examined pre-study respiratory medication use. Although in an unadjusted analysis we demonstrated that subjects with a history of asthma were more likely to be using inhaled corticosteroids, this difference did not remain significant when adjusted for FEV_1_. As inhaled corticosteroids are often used in the treatment of moderate to severe COPD as well as asthma, examining a history of this medication use may not be helpful in distinguishing subjects with asthma. We limited our inclusion to subjects diagnosed with asthma before the age of 40 in order to identify subjects whose asthma diagnosis preceded their COPD diagnosis and to exclude subjects who might have other respiratory diseases confused with asthma.

In conclusion, we demonstrate that subjects with COPD and asthma demonstrate unique clinical features including an increase in respiratory exacerbations. These findings have implications for disease management and treatment. Improved monitoring and prevention of exacerbations in patients with COPD and asthma may improve quality of life and potentially survival.

## Abbreviations

6MWT: six-minute walk test; AHR: Airway hyperresponsiveness; ATS: American Thoracic Society; BMI: Body Mass Index; BODE: Body mass index, airflow Obstruction, Dyspnea, and Exercise capacity; CI: Confidence Interval; COPD: Chronic Obstructive Pulmonary Disease; COPDGene: Genetic Epidemiology of COPD Study; HU: Hounsfield units; FEV_1_: forced expiratory volume in 1 second; FVC: forced vital capacity; GOLD: Global Initiative for Chronic Obstructive Lung Disease; OR: Odds Ratio; Pi10: square root wall area of a hypothetical airway of 10 mm internal perimeter; SGRQ: St. George's Respiratory Questionnaire.

## Competing interests

The authors declare that they have no competing interests.

## Authors' contributions

Study conception and design: MH, CH. Data collection: MH, ES, RB, NH, JS, BM, JC, CH. Data analyses and statistical support: CH, MH. Manuscript writing and editing: MH, ES, RB, NH, BM, JC, CH. MH takes full responsibility for the work represented in this manuscript.

All authors have read and approved the final manuscript.

## Disclosures

Dr. Silverman has received grant support and consulting fees from GlaxoSmithKline for studies of COPD genetics and has received honoraria and consulting fees from AstraZeneca. None of the other authors has reported conflicts of interest.
